# Supplementary material for: Identification of photoperiod-induced specific miRNAs in the adrenal glands of Sunite sheep (Ovis aries)
Source: Front Vet Sci. 2022 Jul 22;9:888207. doi: 10.3389/fvets.2022.888207 (PMC9354845; doi:10.3389/fvets.2022.888207)
Supplement: Supplementary file 1 [file Data_Sheet_1.ZIP › Supplementary file/miRDeep2 file.zip!/miRDeep2 file/Know_Structure/SPLP42A3/oar-mir-487b.pdf]

miRBase precursor : oar-mir-487b
Total read count : 1823
oar-miR-487b-5p read count : 60
oar-miR-487b-3p read count : 1761
remaining reads : 2

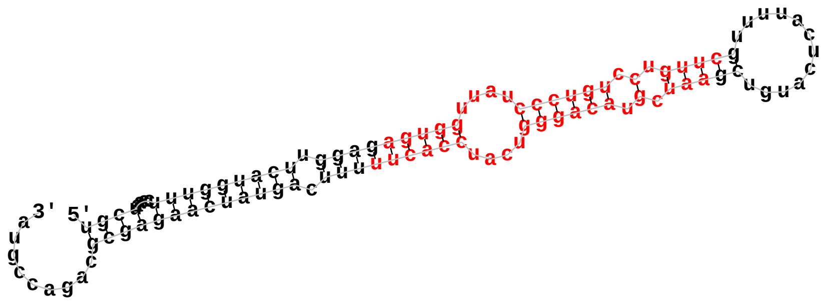

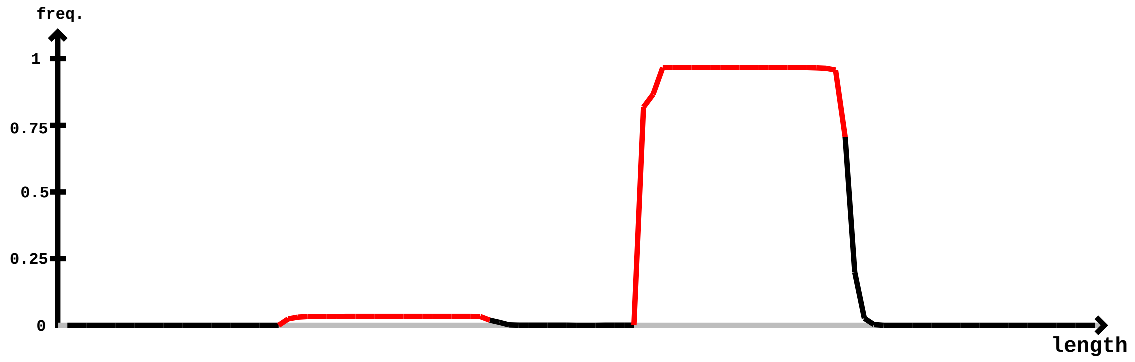


```
                                                               oar-miR-487b-3p
                     oar-miR-487b-5p
5'- ugcuaaccuuugguacuuggagagugguuaucccuguccuguucguuuuacucaugucgaaucguacagggucauccacuuuuucaguaucaagagcgcagaccgua   -3'    exp
    .(((....(((((((((.(((((((((....((((((.(.(((((............))))).).))))))....))))))))).))))))))))))..........    reads      mm         sample
    ......................agugguuaucccuguccugu.................................................................    1          0          seq
    ......................agugguuaucccuguccuguu................................................................    23         0          seq
    ......................agugguuaucccuguccuguuc...............................................................    9          0          seq
    ......................agugguuaucccuguccuguucg..............................................................    9          0          seq
    ......................agugguuaucccuguccuguucgu.............................................................    2          0          seq
    .......................gugguuaucccuguccuguu................................................................    2          0          seq
    .......................gugguuaucccuguccuguuc...............................................................    4          0          seq
    .......................gugguuaucccuguccuguucg..............................................................    6          0          seq
    ........................ugguuaucccuguccuguuc...............................................................    2          0          seq
    ........................ugguuaucccuguccuguucg..............................................................    2          0          seq
    ............................uaucccuguccuguucguuuuacu.......................................................    1          0          seq
    .......................................................gucgaaucguacagggucauccacu...........................    1          0          seq
    ...........................................................aaucguacagggucaucc..............................    2          0          seq
    ...........................................................aaucguacagggucaucca.............................    3          0          seq
    ...........................................................aaucguacagggucauccac............................    12         0          seq
    ...........................................................aaucguacagggucauccacu...........................    449        0          seq
    ...........................................................aaucguacagggucauccacuu..........................    884        0          seq
    ...........................................................aaucguacagggucauccacuuu.........................    139        0          seq
    ...........................................................aaucguacagggucauccacuuuu........................    1          0          seq
    ............................................................aucguacagggucauccacu...........................    8          0          seq
    ............................................................aucguacagggucauccacuu..........................    25         0          seq
    ............................................................aucguacagggucauccacuuu.........................    46         0          seq
    ............................................................aucguacagggucauccacuuuu........................    9          0          seq
    .............................................................ucguacagggucauccacu...........................    15         0          seq
    .............................................................ucguacagggucauccacuu..........................    130        0          seq
    .............................................................ucguacagggucauccacuuu.........................    33         0          seq
    .............................................................ucguacagggucauccacuuuu........................    5          0          seq
```
